# Tailored Therapy Versus Empiric Chosen Treatment for *Helicobacter pylori* Eradication: A Meta-Analysis

**DOI:** 10.1097/MD.0000000000002750

**Published:** 2016-02-18

**Authors:** Han Chen, Yini Dang, Xiaoying Zhou, Bingtuan Liu, Shiyu Liu, Guoxin Zhang

**Affiliations:** From the Department of Gastroenterology, The First Affiliated Hospital of Nanjing Medical University, First Clinical Medical College of Nanjing Medical University, Nanjing, China.

## Abstract

Although various regimens are empirically accepted for *Helicobacter pylori* eradication, the efficacy might be declined by multiple individual factors. The necessity of a personalized eradication therapy still remains controversial. The aim of the study was to compare tailored therapy with empiric chosen regimens.

Databases of PUBMED, EMBASE, and MEDLINE were searched for eligible studies, published up to October 2015. All relevant controlled clinical trials were included. A random-effect model was applied to compare pooled relative risk (RR) with related 95% confidence intervals (CIs).

Thirteen controlled clinical trials integrating 3512 participants were assessed. Overall, the pooled eradication rates of tailored groups were higher than those of empiric ones (intention-to-treat: RR = 1.16, 95% CI 1.10–1.22; preprotocol: RR = 1.14, 95% CI 1.08–1.21). In subgroup analysis, tailored therapy was superior to 7-day standard triple therapy (RR = 1.22, 95% CI 1.16–1.29) and bismuth-quadruple therapy (RR = 1.14, 95% CI 1.07–1.22) on eradication rates; first-line tailored therapy achieved higher eradication rates than first-line empirical regimens (pooled RR = 1.18, 95%CI 1.14–1.22), whereas tailored rescue regimen showed no difference with empirical ones (pooled RR = 1.16, 95% CI 0.96–1.39). Moreover, among different tailored designs, susceptibility-guided tailored therapy obtained higher eradication rates than empiric groups, independent of CYP2C19 genotype detection (with CYP: RR = 1.16, 95% CI 1.09–1.23; without CYP: RR = 1.14, 95% CI 1.01–1.28). Both molecular test-based and culture-based tailored groups were better on eradication rates than empiric groups (molecular: RR = 1.23, 95% CI 1.11–1.35; culture: RR = 1.13, 95% CI 1.06–1.20).

Compared with empiric chosen treatments, tailored therapy is a better alternative for *H pylori* eradication.

## INTRODUCTION

Since the discovery of *Helicobacter pylori* in 1982, research has been conducted over decades to explore the optimal eradication strategy.^1–3^ According to Kyoto global consensus report, *H pylori*-induced gastritis is classified into the category of infectious disease.^4^ However, the strategy of *H pylori* eradication is difficult to follow the common treatment protocols of most infectious diseases. This is largely ascribed to the unavailability of susceptibility testing for *H pylori* in routine clinical laboratory.^1,5^ Consequently, clinicians usually choose antibiotics empirically in an eradication therapy. Nevertheless, due to the growing tendency of antimicrobial resistance, the unconditional use of standard triple therapy is reported to be obsolete.^5,6^ Although other empiric regimens (e.g., bismuth-quadruple therapy [BQT], sequential therapy) are currently recommended, the effectiveness is still controversial. Actually, many individual factors may compromise the eradication success. These factors include antibiotic resistance pattern, individual genetic morphology, past history of medicine, tolerance of treatment, and also personal compliance.^1,2^ Hence, a precisely targeted regimen is allowed for *H pylori* eradication. Under this situation, there is an emerging trend towards an individualized eradication therapy which is aimed to achieve the optimal drug responses.^3,5,7,8^

During the past decade, the pretreatment susceptibility testing was performed by some studies to avoid antibiotic resistance.^6^ There are mainly 2 types of test methodologies: genotype detection and phenotype identification. The genotypic detection refers to molecular tests (e.g., real-time PCR, fluorescent in situ hybridization) by using samples such as stools and gastric biopsy specimens. The phenotypic identification stands for traditional antimicrobial susceptibility testing (e.g., E-test, ager dilution method) through culture of *H pylori* strains.^9,10^ However, antibiotic resistance is not the only factor to affect the drug effectiveness. Recently, proton pump inhibitor (PPI), whose metabolism depends on CYP2C19-catalyzed reaction, has also been reported to exert influence on therapeutic efficacies.^11,12^ Consequently, new personalized therapies are emerging by adding the detection of CYP2C19 genotype within a tailored design.

Currently, there are merely a few publications of literature reviews for assessing the efficacy of tailored therapies. Therefore, we conducted a meta-analysis to compare tailored eradication therapy with empirical regimens on therapeutic effectiveness of *H pylori* eradication.

## METHODS

### Information Sources and Search Strategy

This meta-analysis was conducted in accordance with PRISMA guidelines. Following the search strategy, one reviewer (CH) conducted a literature search on PubMed, EMBASE, and MEDLINE database by using the following terms: (((((((((tailored therapy) OR tailored eradication) OR tailored treatment)) OR (((personalized eradication) OR personalized therapy) OR personalized treatment)) OR (((pretreatment susceptibility tests) OR susceptibility-based treatment) OR susceptibility-guided)) OR (((cyp2c19 genotype) OR cyp2c19 polymorphism) OR genetic polymorphism)) OR ((((IL-1) OR interleukin-1) OR virulence factors) OR BMI))) AND ((((((*Helicobacter pylori*) OR *H.pylori*) OR *H. pylori*)))). The consent procedure and study protocol were approved by the Medical Institutional Ethical Committee of first affiliated hospital of Nanjing Medical University.

### Eligibility Criteria

All original articles, published up to October 2015, which compared the eradication efficacy between tailored and empiric regimens, were included in this meta-analysis. All studies were published as full articles. The abstracts of these articles were carefully screened by 2 independent reviewers (CH and DYN). Clinical controlled trials were primarily considered. Retrospective studies, case reports, and also other clinical trials without controlled therapeutic groups were all excluded. In addition, the eligible studies should include the accessible data of successful eradication rates in both tailored and empirical groups. Patients meeting the following criteria were excluded: history of medicine within previous 4 weeks; previous history of gastrointestinal malignancy; previous gastric or esophageal surgery histories; severe infectious diseases or systemic disorders, such as severe organ dysfunction; and alcohol abuse or pregnancy or under lactation.

### Data Collection Process

The first reviewer (CH) read the titles and abstracts of each article and then obtained preliminarily eligible studies. The second reviewer (DYN) screened these papers based on eligibility criteria. Reference lists of relevant publications were checked for potentially eligible studies. Contacts were made by e-mails to the authors for any requirements of missing data among eligible studies. Discrepancies were resolved by consensus between the 2 reviewers. Data extraction process was conducted by the first reviewer (CH) and then a further check was made by 3 other reviewers (ZXY, LBT, and LSY).

### Data Items

The following information was extracted in each study: baseline demographics variables (year and country of publication, study design, mean age, sex, and sample size); diagnostic tests of *H pylori* infection; treatment regimens of both tailored and control groups (regimen, dosage, and dose interval); the number of patients in each group who are successfully treated; the eradication rates in both tailored and control arms, side effects during or after eradication (if any trial evaluated); and the cost of each therapy (if any trial calculated).

### Risk of Bias in Individual Studies

**FIGURE 2:**
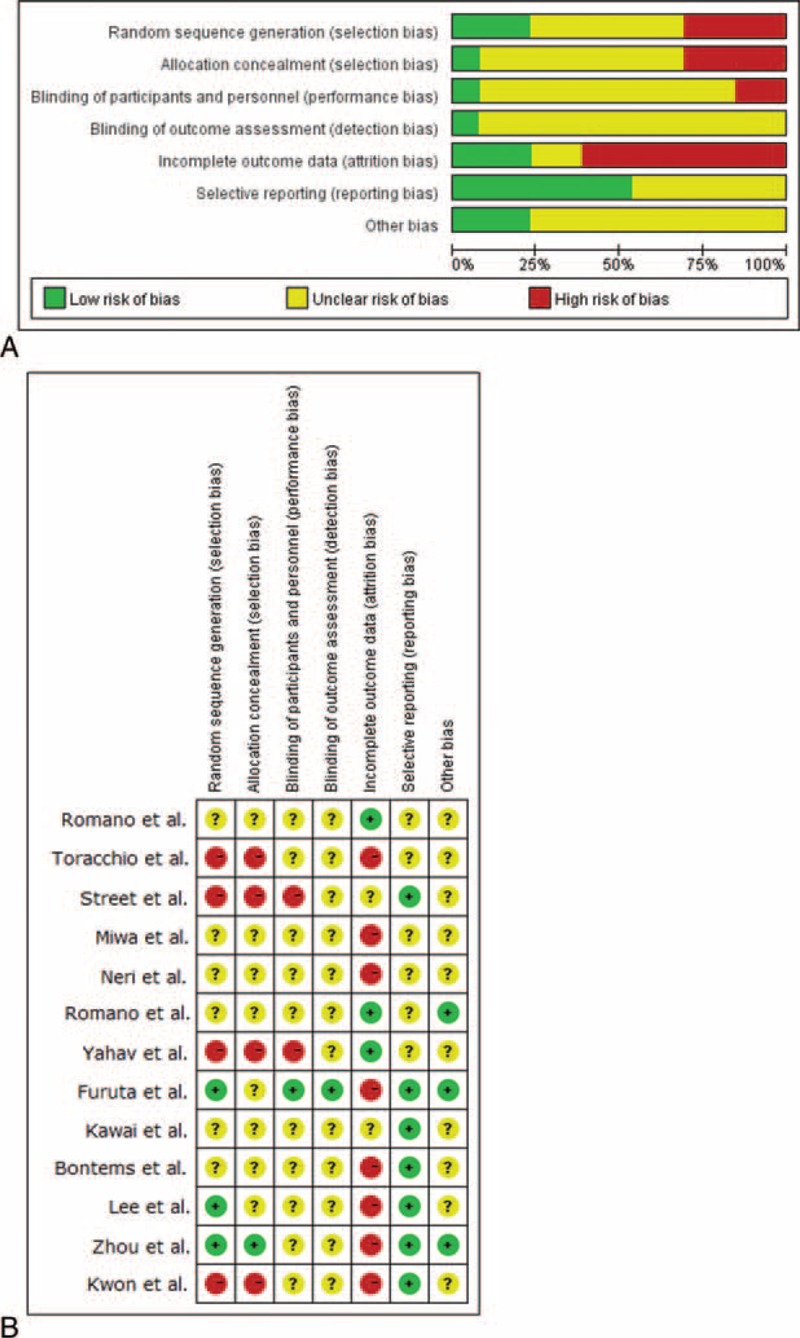
A, Risk of bias graph: reviewer's judgments about each risk of bias item presented as percentages across all included studies. B, Risk of bias summary: reviewer's judgments about each risk of bias item in each study. (+) = low risk of bias, (?) = unclear, (−) = high risk of bias.

The Cochrane Tool of Bias was applied to ascertain the validity of eligible randomized trials. All studies were evaluated by 2 independent reviewers (CH and ZXY) with adequate reliability in determining the following domains: the adequacy of randomization and concealment of allocation, blinding of participants, personnel and outcome assessors, the extent of loss to follow-up, the assessment of selective outcome reporting, and other sources of bias. Discrepancies were resolved by consensus between the 2 reviewers (Figure 2A and B).

### Risk of Bias Across Studies

**FIGURE 5:**
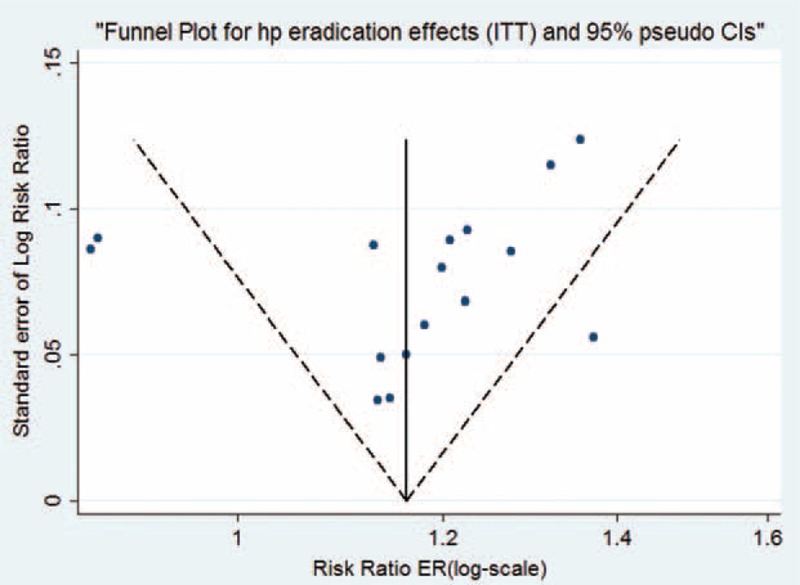
Funnel plot analysis of 13 studies. Statistical analysis confirmed no evidence of publication bias.

Statistical heterogeneity across the studies was assessed visually with Begg funnel plot (Figure 5). Harbord modified test was also applied.

### Statistical Analyses

The meta-analyses were performed by computing relative risks (RRs) using random-effects model. Quantitative analyses were performed on an intention-to-treat (ITT) and preprotocol (PP) basis, with RR and related 95%confidence intervals (CIs) for each. Meta-regression and subgroup analysis were performed for additional analysis.

## RESULTS

### Study Selection and Characteristics

**FIGURE 1:**
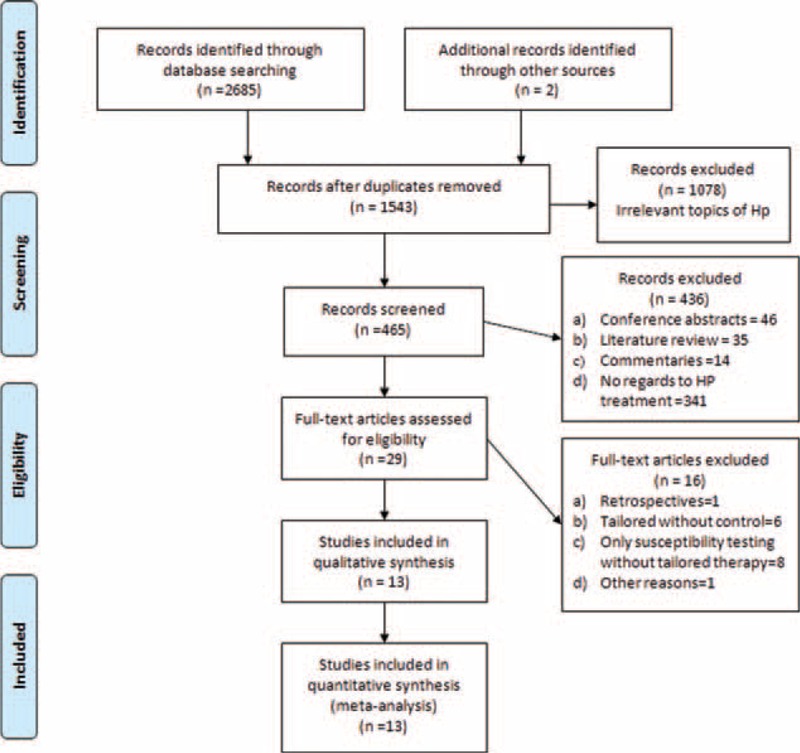
Flow chart of studies.

Figure 1 details the procedure of study selection in the flow chart. Thirteen studies^13–25^ were qualified in this meta-analysis. Tables 1 and 2  summarize the baseline characteristics. A total 3512 participants received treatments of *H pylori* eradication. Among them, 1295 participants received tailored regimens, whereas 2217 received empirical treatments. Ten studies were randomized control trials^13–18,20,22-24^ and 3 were nonrandomized controlled clinical trials.^19,21,25^ In terms of areas, 7 studies^13,18,20-23^ were reported in Asia and 6 studies^14-17,19,24^ were reported from Europe. Moreover, 3 studies^18,23,25^ set 2 different control groups, respectively, which were labeled as group a and b in our study (e.g., Lee a and Lee b). The quality of publication evaluated was of medium-to-low quality evidence and only 1 study had low risk of bias. Both Begg funnel plot (*P* = 0.893) and Harbord modified test (*P* = 0. 0089) indicate no evidence of heterogeneity across the studies (Figure 5).

**TABLE 1:**
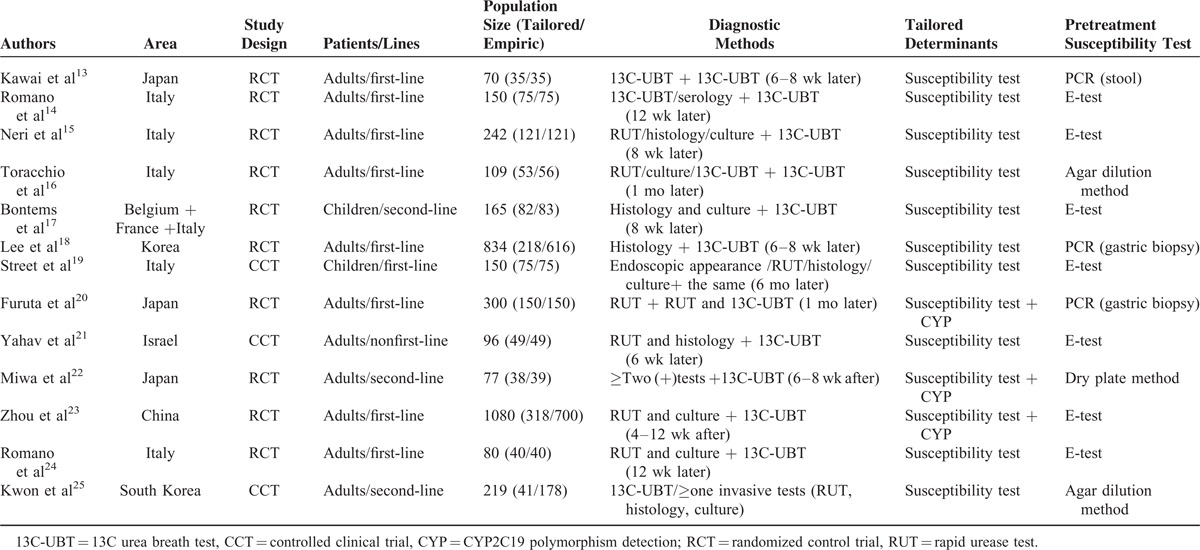
| Baseline Characteristics of Included Studies

**TABLE 2:**
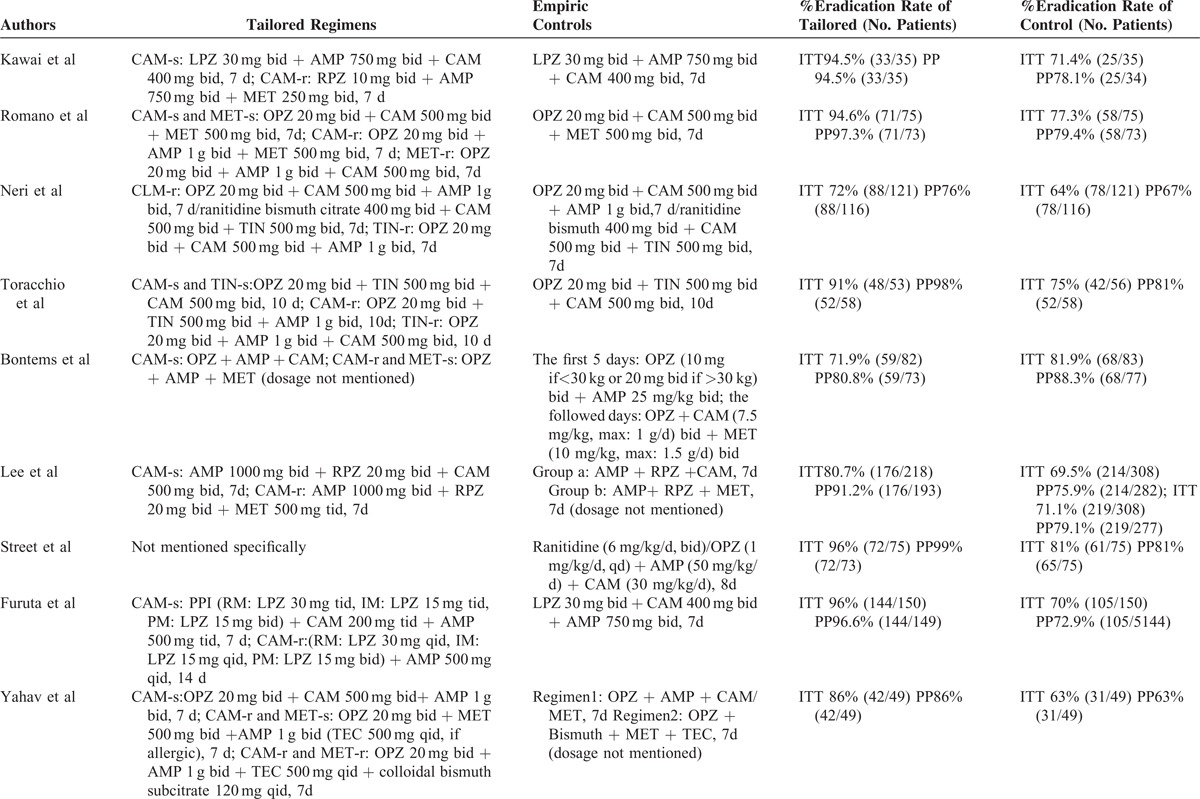
| Major Regimens and Eradication Rates of Included Studies

**TABLE 2 (Continued):**
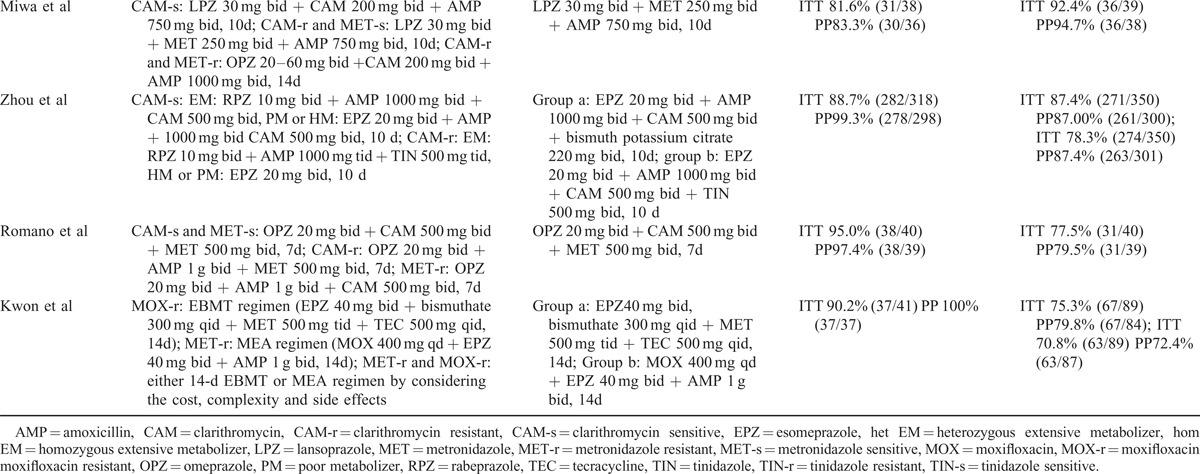
| Major Regimens and Eradication Rates of Included Studies

### Eradication Rate

**FIGURE 3:**
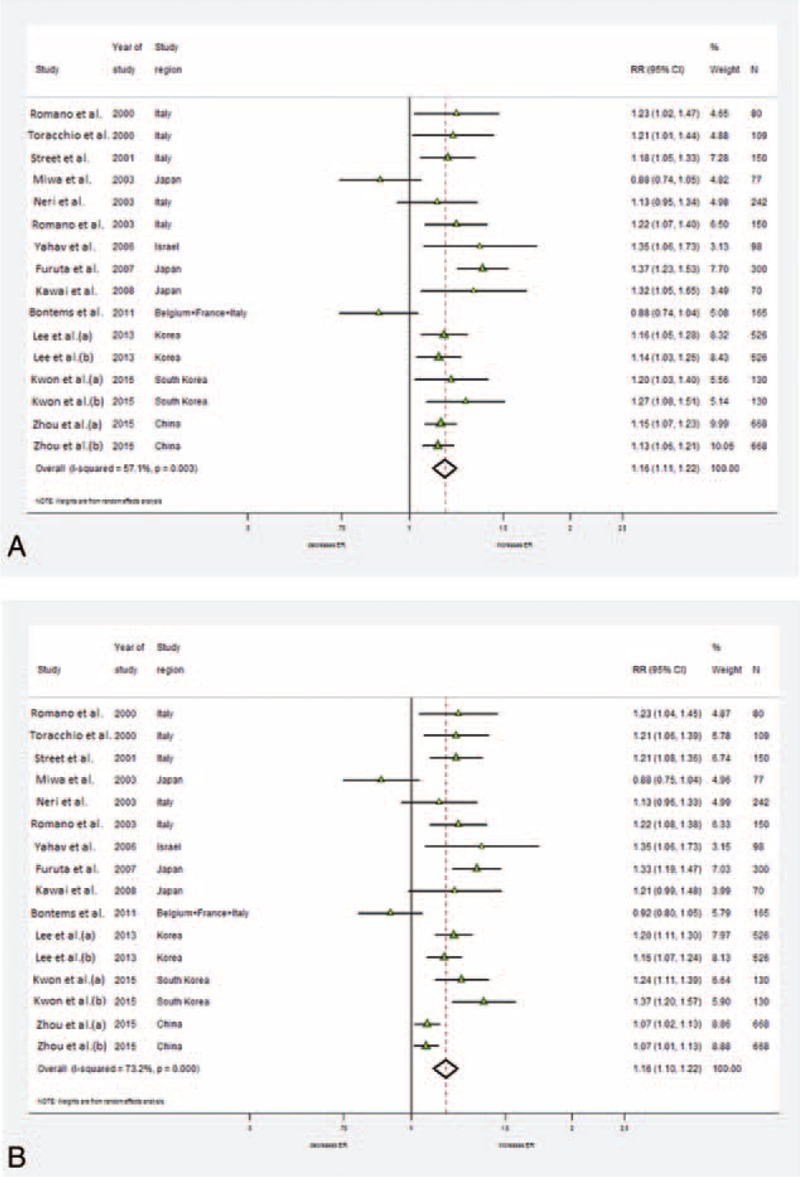
Forest plot of tailored therapy versus empiric treatments on eradication rates by intention-to-treat (ITT) analysis in (A) and by preprotocol (PP) analysis in (B). A random-effect model was used. Significant heterogeneity was shown among the studies in both ITT (I^2^ = 57.1%, *P* = 0.003) and PP (I^2^ = 73.2%, *P* = 0.000).

In 13 trials, data of eradication rates were available in 3246 participants (266 were lost to follow-up). The pooled RR of ITT in tailored groups over control groups was 1.16 (95% CI 1.11–1.22) and the pooled RR of PP was 1.16 (95% CI 1.10–1.22), both with the evidence of high heterogeneity (ITT: I^2^ = 57.1%, *P* = 0.003; PP: I^2^ = 73.2%, *P* = 0.000) (Figure 3A and B). Meta-regression demonstrates no significant difference of study design (*P* = 0.345) and area (*P* = 0. 0.600), pediatric/adult population (*P* = 0.641), and sex (*P* = 0.577).

### Subgroup Analysis

Tailored therapy shows its superiority over empirical treatment in both Asia (pooled RR = 1.18, 95% CI 1.11–1.25) and Europe (pooled RR = 1.14, 95% CI 1.03–1.25).

### Types of Tailored Regimens

**FIGURE 4:**
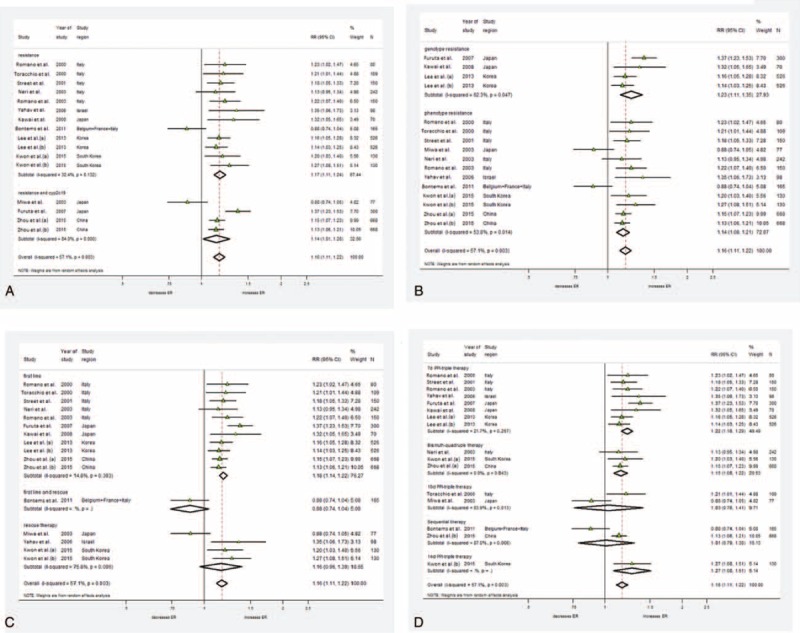
Forest plot of subgroup analysis. A, Among different types of tailored groups, both regimens tailored by antibiotic resistance (RR = 1.17, 95% CI 1.11–1.24) and regimens tailored by antibiotic resistance and CYP2C19 detection (RR = 1.14, 95% CI 1.01–1.28) achieved higher eradication rates than empiric regimens. Significant heterogeneity was shown among the studies in both subgroups. B, There were higher eradication rates in both genotypic (RR = 1.23, 95% CI 1.11–1.35) and phenotypic (RR = 1.14, 95% CI 1.08–1.21) detection of antibiotic resistance of tailored groups than empiric groups. Significant heterogeneity was shown among the studies in both subgroups. C, First-line tailored therapy achieved higher eradication rates than first-line empirical regimens (pooled RR = 1.18, 95% CI 1.14–1.22). There is no significant difference in eradication rates between tailored rescue regimen and empirical rescue ones (pooled RR = 1.16, 95% CI 0.96–1.39). No heterogeneity was shown among first-line tailored groups, whereas significant heterogeneity was shown among rescue groups. D, Among empiric groups, the eradication rates were lower in 7-day triple therapy (RR = 1.22, 95% CI 1.16–1.29) and bismuth-quadruple therapy (RR = 1.14, 95% CI 1.07–1.22) than in tailored ones. Either eradication rates of 10-day triple therapy (RR = 1.03, 95% CI 0.76–1.41) or of sequential therapy (RR = 1.01, 95% CI 0.79–1.30) show no difference from eradication rates of tailored groups. No heterogeneity was shown in both 7-day triple group and bismuth-quadruple group, whereas significant heterogeneity was shown in 10-day triple and sequential groups. CI = confidence interval, RR = relative risk.

Pretreatment susceptibility testing and CYP2C19 polymorphisms were 2 main determinants for designing tailored therapy. Ten tailored regimens^14-17,19,21-25^ were designed according to pretreatment susceptibility testing (pooled RR = 1.17, 95% CI 1.11–1.24). Three other studies^13,18,20^ advanced their susceptibility-guided therapy by additionally adjusting their PPI administration (either by dosage adjustments or by changing drugs) on the basis of CYP2C19 polymorphism (pooled RR = 1.14, 95% CI 1.01–1.28). The analytical results indicate that both types of tailored therapy are better than empirical treatments in achieving higher eradication rates (Figure 4A).

### Methods of Antibiotic Susceptibility Testing

All 13 tailored trials applied pretreatment susceptibility tests for detecting individual antibiotic resistance patterns. In 3 studies,^20,22,23^ genetic resistance of antibiotics were detected by molecular methods (pooled RR = 1.23, 95% CI 1.11–1.35). Ten other studies performed traditional culture-based tests in detecting phenotype resistance patterns (pooled RR = 1.14, 95% CI 1.08–1.21); the pooled results demonstrate that susceptibility-guided tailored therapies achieved higher eradication rates than empirical regimens by using either molecular-based or traditional culture-based test (Figure 4B).

### First-Line and Nonfirst-Line Tailored Therapy

Nine studies designed first-line tailored therapy, whereas 3 studies^21,22,25^ applied salvage tailored therapy. One trial^17^ performed tailored regimen as both first-line and rescue therapy. The pooled results indicate that first-line tailored therapy obtained higher eradication rates than first-line empirical regimens (pooled RR = 1.18, 95% CI 1.14–1.22). There is no significant difference in eradication rates between tailored rescue regimen and empirical rescue ones (pooled RR = 1.16, 95% CI 0.96–1.39) (Figure 4C).

### Different Empiric Regimens

In total, there were 5 different empiric regimens in 16 groups. In 7 studies,^13,14,18-21,24^ participants from empiric groups received 7-day standard triple therapy. Two trials applied the 10-day therapeutic duration. BQT was used in 3 trials^15,23,25^ (Zhou et al group a and YH Kwon et al. group a). Two studies^17,23^ (Zhou et al, group a) selected sequential therapy, and 1 trial applied 14-day moxifloxacin-containing triple regimen^25^ (Kwon et al, group b). These results show that tailored therapy achieved higher eradication when compared with 7-day standard triple therapy (pooled RR = 1.22, 95% CI 1.16–1.29), BQT (pooled RR = 1.15, 95% CI 1.08–1.22), and 14-day moxifloxacin-containing triple regimen (pooled RR = 1.27, 95% CI 1.08–1.51). Unexpectedly, tailored therapy shows no significant differences in eradication rates with 10-day-triple therapy (pooled RR = 1.03, 95% CI 0.76–1.41) and sequential therapy (pooled RR = 1.01, 95% CI 0.79.–1.30) (Figure 4D).

## DISCUSSION

### Summary of Evidence

This is the first meta-analysis in evaluating the potential therapeutic efficacy of tailored therapy in *H pylori* eradication. Our meta-analysis has 5 principal findings: overall, tailored therapy was more efficacious than empiric one; higher eradication rates were achieved than those of empiric regimens in a susceptibility-based tailored therapies, irrespective of CYP2C19 genotype polymorphism; both culture-based and molecular-based tailored therapy obtained good therapeutic efficacies; tailored therapy achieved better effectiveness than 7-day standard triple therapy and BQT; the first-line tailored therapy is better than empiric treatments, whereas tailored rescue therapy did not perform better than empiric ones.

Here, we defined tailored therapy as a precisely targeted *H pylori* eradication therapy which emphasizes on predicting individual drug responses before treatment.^1,6-8,13-25^ Actually, tailored therapies are diversified. Different adjectives have been used to describe it as tailored, personalized, individualized, culture-based, pharmacogenetic-based, and susceptibility-guided.^13-25^ This attributes to the fact that multiple factors will affect the final eradication success.^1,6^ These factors include antibiotic resistance, dosing of acid inhibitory drugs, genotypes of drug-metabolizing enzymes, drug transporters, inflammatory cytokines (i.e., interleukin [IL]-1β), one's past medical history, treatment tolerance, and also personal compliance.^6,11,26^ Rationally, an eradication treatment should be evidence-based.^2^ Since the drug response varies from person to person, patients will benefit from an individualized treatment as precisely as possible. However, when considering the cost and feasibility, it is difficult to include all individual factors into a tailored design. Hence, it is better to identify the main influential factors as the major tailored determinants.

Antibiotic resistance is considered to be one of the main reasons for eradication failure.^27-29^ Thus, it is considered as a major tailored determinant by most tailored trials. Importantly, our result challenges the necessity of performing traditional susceptibility tests within a tailored therapy. Although traditional methodologies are useful in determining phenotypic resistance patterns of antibiotics,^10^ they are rarely available in routine clinical practice. There are several reasons: first, it is fastidious and time-consuming to grow *H pylori* in culture^30^; second, there is no standard method for the interpretation of susceptibility^17^; and third, the in vitro test might not reflect the actual levels of antibiotics in the gastric lumen in which there is possible pH influence on antimicrobial activity.^21^ Consequently, such tests are usually considered within a salvage therapy after multiple treatment failures.^1,3,27^ Currently, new molecular tests begin to emerge, allowing clinicians to obtain evidence of antibiotic resistance without culture procedures. Some publications reported that therapies tailored by molecular tests achieved higher success rates than those by traditional culture-based tests.^26-28^ Actually, molecular tests are advantageous: firstly, they have simple procedures and are time-saving; moreover, clinicians can easily obtain stool samples or gastric specimens through endoscopic biopsies.^10^ Hence, it is worthwhile to further estimate the value of molecular tests for antimicrobial resistance.

The second tailored determinant is the individual CYP2C19 genotype. In this study, the role of CYP2C19 polymorphisms detection is challenged in a susceptibility-guided tailored therapy. A literature review of tailored eradication therapy indicates that a tailored treatment designed according to pharmacogenomics and antimicrobial susceptibility achieves an eradication rate exceeding 95%, irrespective of eradication history, and overcomes differences among CYP2C19 genotypes.^12^ However, our results show that CYP2C19 detection may be less clinically significant when antibiotic resistance has already been taken into account within a tailored design. Although rapid metabolizers (RMs) are reported to have decreased eradication rates than intermediate/poor ones,^11,12^ the influence of CYP genotype in RM is probably overcome by increasing PPI dosing or by administrating advanced PPI such as rabeprazole or esomeprazole, which rarely metabolizes through CYP2C19 pathway.^11^ Considering that PPI administration varies in trials, more randomized clinical trials are needed for evaluating the role of CYP2C19 detection on improving eradication rate in tailored therapies.

The next assessment in our meta-analysis is the efficacy of tailored therapies as the first-line or rescue regimens. Currently, tailored therapy is not routinely applied as a first-line eradication treatment.^1^ According to the Maastricht Consensus Conferences, the antibiotic susceptibility testing before antibiotic therapy is suggested after the failure of second-line treatment.^28^ Nevertheless, in our analysis, better eradication rates were achieved in most first-line tailored regimens than in the empiric groups, indicating the potential value of tailored regimen as an alternative first-line eradication choice. However, when it comes to rescue tailored therapy, the advantage is not so obvious. Here, the pooled results are mainly influenced by 1 trial conducted by Miwa et al concluding that susceptibility testing is not necessarily required before second-line therapy if the first-line treatment has been performed by PPI/AC regimens.^21^ Since the efficacy of second-line treatment is greatly affected by the previous first-line regimen choice,^31,32^ it is possible that other individual factors, such as previous medicine or personal compliance, should also be considered into a tailored design to achieve better effectiveness. As there is significant heterogeneity among the 3 rescue tailored groups, more randomized trials are needed to further assess the potential value of tailored rescue therapy.

Furthermore, we compared tailored therapy with different commonly recommended empiric treatments. Here, our result is consistent with the current opinion that 7-day standard triple therapy is obsolete mainly due to clarithromycin resistance.^29,33,34^ Since pretreatment susceptibility tests would help overcome antibiotic resistance, tailored therapy is superior to standard triple therapy in eradication rate. However, the advantage of tailored therapy is undermined when the duration of triple therapy is prolonged to 10 days. The explanation is probably that increasing duration will increase the drug effectiveness to overcome antibiotic resistance in standard triple therapy.^35^ In this sense, the advantages of tailored therapy are still controversial. Meanwhile, we discovered that tailored therapy is superior to BQT in eradication improvement. The possible explanation is that BQT is advantageous by partially overcoming the resistance to major antibiotics such as clarithromycin or levofloxacin,^1,36^ but it is less targeted when compared with tailored therapy in getting precise evidence of antibiotic resistance patterns on individual levels. Therefore, for its evidence-based characteristics, tailored therapy is better than BQT in individual therapeutic precision.

### Limitations

There are several limitations in this meta-analysis. Firstly, we are unable to analyze the side effects for further investigating the feasibility of tailored regimens. Most of the trials merely focused on eradication rates, and only 4 trials provided data of side effects. Secondly, we failed to include the cost in both groups. Although 2 trials demonstrated that tailored therapy is more cost-saving than standard triple therapy (saving $5 and $12 on average, respectively), there were still insufficient data to show whether tailored therapy could be more cost effective than other popular empirical regimens. Thirdly, the 3 trials were not randomized, which might have affected the validity of the overall findings. Furthermore, due to the small sample sizes of clinical trials included in our meta-analysis, large-scale randomized clinical trials are urgently warranted with regards to comparison of therapeutic efficacy between tailored regimens and different empiric ones.

## CONCLUSIONS

In summary, compared with empiric chosen regimen, tailored therapy is a better alternative for *H pylori* eradication. It is clinically significant to promote broader assessments of tailored therapy compared with different empirical treatments worldwide. We also suggest further research regarding more therapeutic innovations customized for specific individuals with *H pylori* infection.
